# Differences in Thyroid Function and Autoantibodies in the Development of Guillain–Barré Syndrome vs. Chronic Inflammatory Demyelinating Polyradiculoneuropathy

**DOI:** 10.3389/fneur.2020.01018

**Published:** 2020-09-10

**Authors:** Yu Tu, Xuan Gong, Guanwen Zeng, Wenyan Zhuo, Zhaoxia Li, Xueying Yu

**Affiliations:** ^1^Zhuhai People's Hospital (Zhuhai Hospital Affiliated With Jinan University), Zhuhai, China; ^2^Beijing Tiantan Hospital, Capital Medical University, Beijing, China

**Keywords:** Guillain–Barré syndrome, chronic inflammatory demyelinating polyradiculoneuropathy, thyroid peroxidase antibody, thyroglobulin antibody, serum total thyroxine

## Abstract

**Background:** Both Guillain–Barré syndrome (GBS) and chronic inflammatory demyelinating polyradiculoneuropathy (CIDP) are neurodegenerative and inflammatory demyelination disorders. Sporadic reports showed that the increased levels of thyroid function and autoantibodies are associated with GBS, CIDP, or both, but no systematic study has been reported. We assessed the differences of thyroid function and autoantibodies between GBS and CIDP in a Chinese cohort.

**Methods:** A total of 256 patients were enrolled in this study. 175 clinically confirmed GBS and CIDP patients were selected. Meanwhile, 81 patients hospitalized for diseases other than GBS or CIDP with mild symptoms were enrolled as a control group. Relevant clinical data, including thyroid function, and autoantibody examinations, were collected for statistical analysis.

**Results:** In the comparison of thyroid function and autoantibody parameters, the levels of total thyroxine (TT4), thyroid peroxidase antibody (TPO-Ab), and thyroglobulin antibody (TG-Ab) in the GBS group were all higher than those in the CIDP and Control groups (*P* < 0.01). The thyroid antibody positive rates in the GBS and CIDP groups were 70.10 and 14.10%, respectively (*P* < 0.01). In the receiver operating characteristic (ROC) curve analysis, TT4, TPO-Ab, and TG-Ab were higher in the GBS group and lower in the CIDP group (*P* < 0.01). To achieve a high specificity of 97–99%, the diagnostic cutoff value of TPO-Ab was higher than 133 IU/mL (Sensitivity: 11.34%) or lower than 0.01 IU/mL (Sensitivity: 9.09%), while the diagnostic cutoff value of TG-Ab was higher than 261.1 IU/mL (Sensitivity: 2.06%) or lower than 0.46 IU/mL (Sensitivity: 11.69%). Multivariate logistic regression analysis showed that the differences in TPO-Ab were statistically significant between GBS patients with TPO-Ab was higher than 133 IU/mL and CIDP patients (*P* < 0.01); the differences in TG-Ab were statistically significant between GBS patients with TG-Ab was higher than 261.1 IU/mL and CIDP patients (*P* < 0.05).

**Conclusion:** The elevation of thyroid autoantibodies was associated with GBS. TPO-Ab higher than 133 IU/mL or lower than 0.01 IU/mL and TG-Ab higher than 261.1 IU/mL or lower than 0.46 IU/mL had high specificity for differentiating between GBS and CIDP; therefore, TPO-Ab and TG-Ab can be used as biomarkers for the differential diagnosis of GBS and CIDP.

## Introduction

Guillain–Barré syndrome (GBS) is an autoimmune peripheral neuropathy characterized by demyelination of peripheral nerves and nerve roots. The clinical manifestation is acute symmetric flaccid limb paralysis, which usually peaks within 4 weeks of disease onset. Respiratory muscle involvement is common, and glucocorticoid treatment alone is not effective in terms of clinical outcomes (1).

Chronic inflammatory demyelinating polyradiculoneuropathy (CIDP) is a disease of the peripheral nervous system (PNS) that is clinically characterized by symmetrical proximal and distal weakness with altered sensation and hyporeflexia/areflexia (2). CIDP exhibits a chronic progression or remission–relapse disease course, and the disease usually peaks 8 weeks after disease onset. Respiratory muscle involvement is rare, and most patients have excellent responses to immunotherapy and corticosteroids (3). Clinically, the pathological characteristics and clinical symptoms of CIDP are similar to those of GBS. Differentiation between CIDP and GBS at the early stage is difficult, as both diseases are easy to misdiagnose.

Thyroid peroxidase antibody (TPO-Ab) and thyroglobulin antibody (TG-Ab) are major indicators for the diagnosis of Hashimoto's thyroiditis (HT). The general belief is that TPO-Ab should be measured to identify autoimmune thyroiditis diseases in a manner similar to HT (4). Bairactaris et al. (5) reported one patient with CIDP and HT who had elevated TPO-Ab and TG-Ab levels. Toudou et al. (6) reported one patient with GBS and HT who had elevated TPO-Ab levels. The serum thyroxine (T4) level has been associated with the incidence and severity of GBS (7). Only sporadic case reports show that GBS, CIDP, and HT are associated, and no clinical study has analyzed all three diseases together. This study retrospectively analyzed thyroid autoantibodies (TPO-Ab and TG-Ab) and thyroid function to confirm the associations between GBS, CIDP, and HT. These measurements were conducted to provide a more theoretical basis for the early differential diagnosis of GBS and CIDP and to prevent clinical misdiagnosis.

## Materials and Methods

### Study Subjects

#### GBS and CIDP Groups

GBS and CIDP patients who were discharged between October 2014 and February 2020 were enrolled. The inclusion criteria were as follows: GBS or CIDP diagnosed at Beijing Tiantan Hospital, Capital Medical University, and confirmed according to Guillain–Barré and Miller Fisher syndromes—new diagnostic classification (8) and the European Federation of Neurological Societies/Peripheral Nerve Society Guideline on management of chronic inflammatory demyelinating polyradiculoneuropathy (3), respectively; an age of 18–83 years; and complete cerebrospinal fluid (CSF) examinations, including CSF cell counts, CSF protein measurement, and 24-h intrathecal IgG synthesis testing; complete electromyography (EMG); thyroid antibody detection; autoimmune antibody detection; and tumor indicator detection. This retrospective study was approved by our institutional ethics committee with a waiver of informed consent. According to the Hughes Functional Grading Scale (HFGS) score (9), HFGS was ≤ 4 at the peak of the disease.

The exclusion criteria were as follows: comorbid diseases affecting brain function, such as stroke, transient ischaemic attack, encephalitis, and subarachnoid hemorrhage; systemic infectious diseases, malignant tumors, severe heart, liver, or kidney diseases, or hematological diseases; sequelae of nervous system diseases precluding determination of the severity of GBS; a history of treated or untreated thyroid disease; the use of drugs affecting thyroid function; a history of hypothalamic and pituitary diseases; and ventilator-assisted ventilation or death due to GBS or CIDP.

#### Control Group

The control group consisted of patients who were discharged between October 2014 and February 2020. The control patients were hospitalized for conditions other than GBS and CIDP with mild symptoms, such as dizziness, primary headache, leukodystrophy, multiple sclerosis, and minor stroke.

### Research Methods

#### Thyroid Functions and Autoantibody Detection

A blood sample (5 mL) was collected from the median cubital vein of each patient in the morning within 48 h of admission. Blood samples were allowed to stand for 30 min and centrifuged at 3,000 r/min for 10 min. autoantibodies were detected within 1 h of centrifugation. The Abbott automatic biochemical immune analyser (Architectcil6200) and original reagent detection and the chemiluminescence immunoassay method were used to detect serum levels of free triiodothyronine (FT3), free thyroxine (FT4), T3, T4, thyroid-stimulating hormone (TSH), TG-Ab, and TPO-Ab. When the autoantibodies concentration of the sample was below the lower detection limit (or above the upper detection limit), the lower detection limit value (or the upper detection limit value) was used.

#### Determination Standards of Abnormal Thyroid Functions

Based on the Clinical Practice Guidelines for Hypothyroidism in Adults:

Cosponsored by the American Association of Clinical Endocrinologists and the American Thyroid Association (4) and the standards used in Beijing Tiantan Hospital, Capital Medical University, the normal values for thyroid functions and autoantibodies were as follows: ① serum total thyroxine (TT4): 69.97–152.52 nmol/L; ② serum FT4: 7.64–16.03 pmol/L; ③ serum total triiodothyronine (TT3): 1.01–2.48 nmol/L; ④ serum FT3: 3.28–6.47 pmol/L; ⑤ serum TSH: 0.49–4.91 μIU/mL; ⑥ serum TG-Ab: 0–4 IU/mL; and ⑦ serum TPO-Ab: 0–9 IU/mL.

#### Statistical Methods

The data were analyzed with SPSS 24.0 statistical software. Measurement data that conformed to or approximated a normal distribution are expressed by x ± s. These means were compared between the groups by the two-independent-sample *t*-test. Non-normal data are expressed as the median and interquartile range and were compared using the Kruskal–Wallis *H*-test. Count data are described as a ratio or percentage (%) and were compared between groups with the χ^2^ test. The GBS or CIDP group assignment was used as the dependent variable, and general patient data that were significantly different between the groups were used as the independent variables for binary multivariate logistic regression analyses. MedCalc19.1 was used to analyse receiver operating characteristic (ROC) curves to evaluate the ability of thyroid functions and autoantibodies to predict GBS and CIDP. *P* < 0.05 was considered statistically significant.

## Results

### Comparison of Basic Data

A total of 256 patients were enrolled in this study. The GBS group (97 cases) included 60 patients with classical acute inflammatory demyelinating polyneuropathy, 19 patients with acute motor axonal neuropathy, 4 patients with acute motor-sensory axonal neuropathy, 11 patients with Miller Fisher syndrome, and 3 patients with acute sensory neuropathy. The CIDP group (78 cases) included 66 patients with classical CIDP, 1 patient with pure motor CIDP, and 11 patients with Lewis-Sumner syndrome. Two acute-onset CIDP (A-CIDP) patients were identified through dynamic patient follow-up and review. There were 81 patients in the control group, and all of them were hospitalized patients with mild disease who did not have GBS or CIDP.

The GBS group had an average age of 49.04 ± 16.19 years, the CIDP group had an average age of 51.67 ± 13.31 years, and the control group had an average age of 34.98 ± 12.22 years. Age in the GBS and CIDP groups was higher than that in the control group (*P* < 0.01), but there was no significant difference between the GBS and CIDP groups (*P* > 0.05). A comparison of the sex distribution showed that the CIDP group included significantly more males than females (χ^2^ = 7.69 and *P* < 0.01). HFGS showed no significant difference between the GBS and CIDP groups (*P* > 0.05; Table 1).

**Table 1:** Comparison among clinical data of the GBS, CIDP, and Control groups, x ± s.

**Subjects**	**All patients**	**Age (years)**	**Sex**		**HFGS**
			**Male**	**Female**	
GBS group	97	49.04 ± 16.19	51	46	3.71 ± 0.52
CIDP group	78	51.67 ± 13.31	57	21	3.85 ± 0.40
Control group	81	34.98 ± 12.22	30	51	0.10 ± 0.30
*F*/*χ^2^*		32.79	20.88		2,088.69
*P*		0.00**	0.00**		0.00**

Differences in sex were statistically significant between the GBS and CIDP groups (χ^2^ = 7.69, P < 0.01), while HFGS showed no significant difference (P > 0.05). Age and HFGS in the GBS and CIDP groups were higher than those in the control group (**P < 0.01).

### Comparison of Thyroid Functions and Autoantibody Parameters

**Figure 1:**
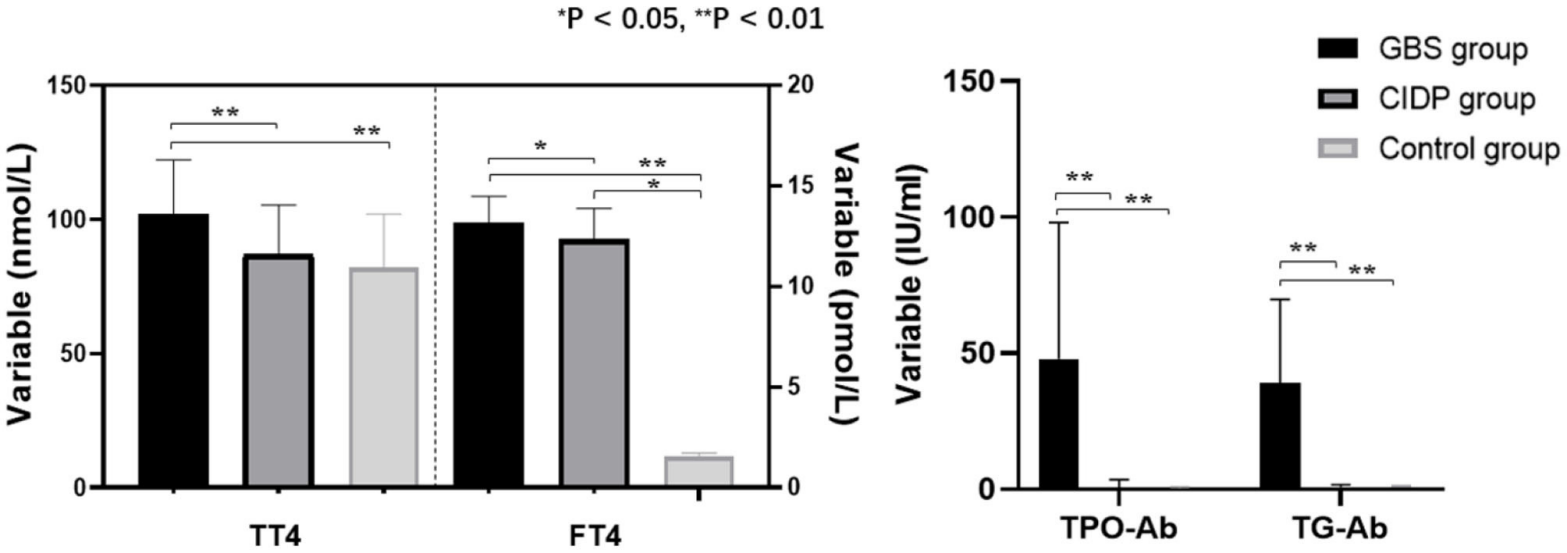
Comparison of thyroid function and autoantibodies parameters among the GBS, CIDP, and Control groups. TT4, FT4, TPO-Ab, and TG-Ab levels were all higher in the GBS group than in the CIDP and Control groups (**P < 0.01, *P < 0.05).

TT4, TPO-Ab, and TG-Ab levels were all higher in the GBS group than in the CIDP and Control groups (*P* < 0.01). FT4 levels were higher in the GBS group than in the CIDP group (*P* < 0.05) and Control group (*P* < 0.01). FT4 levels were higher in the CIDP group than in the control group (*P* < 0.05). TT3 was lower in the GBS group than in the CIDP and Control groups (*P* < 0.05). The differences in TSH and FT3 between the three groups did not reach statistical significance (*P* > 0.05). The comparisons of the above meaningful indicators are depicted in Table 2 and Figure 1.

**Table 2:** Comparison of thyroid function and autoantibodies parameters between GBS and CIDP groups M (*P25, P75*).

**Subjects**	**TT3 (nmol/L)**	**TT4 (nmol/L)**	**TSH (μIU/mL)**	**FT3 (pmol/L)**	**FT4 (pmol/L)**	**TPO-Ab (IU/mL)**	**TG-Ab (IU/mL)**
GBS group	1.31 (1.07, 1.57)	101.62 (84.50, 122.20)	1.32 (0.88, 2.16)	4.36 (3.85, 4.77)	13.12 (11.83, 14.49)	47.91 (0.99, 98.00)	39.16 (1.67, 69.78)
CIDP group	1.45^*^ (1.27, 1.58)	86.94^**^ (77.97, 105.41)	1.55 (0.96, 2.78)	4.38 (3.95, 4.78)	12.32 (11.52, 13.88)	0.44^**^ (0.21, 3.62)	0.90^**^ (0.84, 1.69)
Control group	1.41^*^ (1.28, 1.56)	82.2^**^ (71.63, 102.03)	1.49 (1.08, 2.55)	4.29 (3.85, 4.72)	11.79^**^^Δ^ (11.03, 13.01)	0.31^**^ (0.13, 0.77)	0.90^**^ (0.90, 1.27)
*P*	0.042	0.000	0.327	0.776	0.000	0.000	0.000

*Compared with the GBS group, Kruskal–Wallis H-test*,

* *P < 0.05*,

** *P < 0.01*.

*Compared with the CIDP group, Kruskal–Wallis H-test*,

Δ *P < 0.05*.

### Comparison of Thyroid Autoantibodies Positive Rates

**Figure 2:**
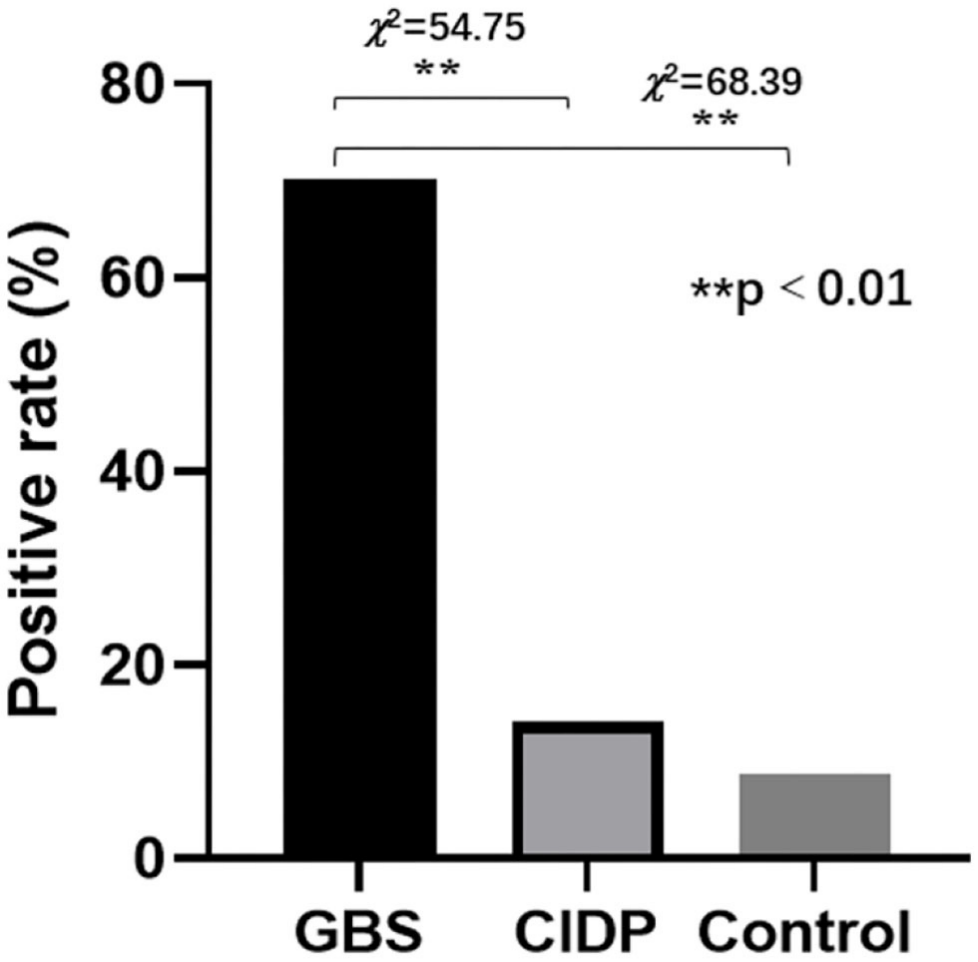
Comparison of thyroid autoantibodies positive rates in the GBS, CIDP, and Control groups. Of the 97 patients in the GBS group, 68 (70.10%) patients were thyroid autoantibody positive. Of the 78 patients in the CIDP group, 11 (14.10%) patients were thyroid autoantibody positive. Of the 81 patients in the control group, 7 (8.64%) patients were thyroid autoantibody positive. The positive rates were significantly different between the GBS and CIDP groups (χ^2^ = 54.75 and **P < 0.01) and between the GBS and Control groups (χ^2^ = 68.39 and **P < 0.01). However, they were not significantly different between the CIDP and Control groups (*P* > 0.05).

The normal values of thyroid functions and autoantibodies referenced by Beijing Tiantan Hospital were used as standards for analyses. Of the 97 patients in the GBS group, 68 (70.10%) patients were thyroid autoantibodies positive. Of the 78 patients in the CIDP group, 11 (14.10%) patients were thyroid autoantibody positive. Of the 81 patients in the control group, 7 (8.64%) patients were thyroid autoantibody positive. The positive rates were significantly different between the GBS and CIDP groups (χ^2^ = 54.75 and *P* < 0.01). Moreover, the positive rates were significantly different between the GBS and Control groups (χ^2^ = 68.39 and *P* < 0.01) although not between the CIDP and Control groups (*P* > 0.05; Figure 2).

### Analyses of ROC Curves of Thyroid Functions and Autoantibodies Parameters

**Figure 3:**
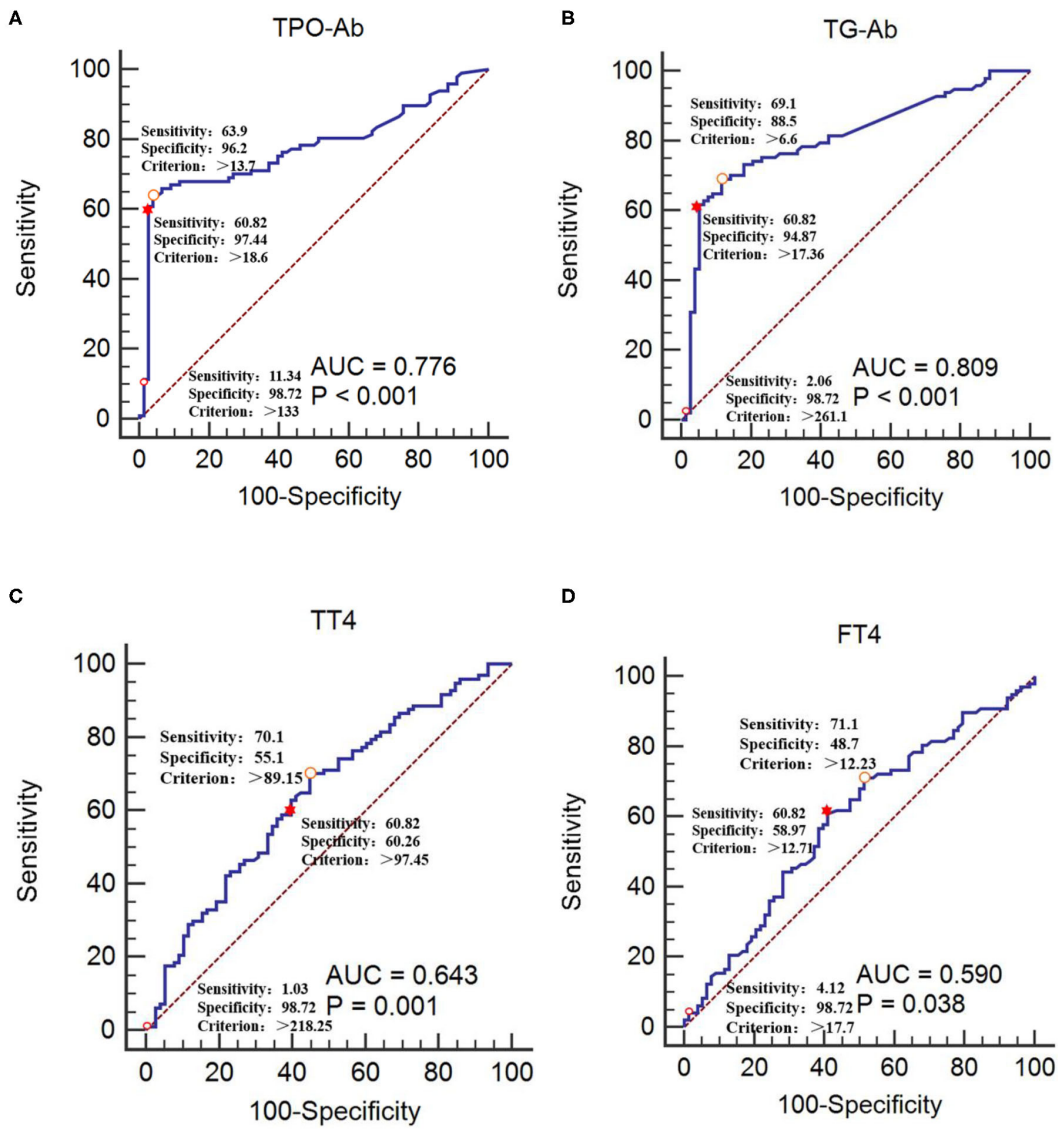
Receiver operating characteristic curves of TPO-Ab, TG-Ab, TT4, and FT4 for GBS occurrence **(A)** TPO-Ab, **(B)** TG-Ab, **(C)** TT4, and **(D)** FT4. The CIDP group was used as the reference group, and the GBS group was used as the measurement group. 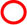 The diagnostic cut-off value corresponds to the maximum point of the Youden index. 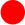 The diagnostic cut-off value corresponds to the maximal specificity. 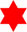 The diagnostic cut-off value corresponds to sensitivity near to 60%.

The CIDP group was used as the reference group, and the GBS group was used as the measurement group. The differences in TT4, FT4, TPO-Ab, and TG-Ab between the GBS and CIDP groups were statistically significant (*P* < 0.05). The diagnostic cut-off value corresponding to the maximum point of the Youden index was obtained. The diagnostic cut-off value of TPO-Ab was 13.7 IU/mL. At this cut-off value, the sensitivity was 63.92%, and the specificity was 96.15%. The diagnostic cut-off value of TG-Ab was 6.6 IU/mL. At this cut-off value, the sensitivity was 69.07%, and the specificity was 88.46%. The diagnostic cut-off value of TT4 was 89.15 nmol/L. At this cut-off value, the sensitivity was 70.10%, and the specificity was 55.13%. The diagnostic cut-off value of FT4 was 12.23 pmol/L. At this cut-off value, the sensitivity was 71.13%, and the specificity was 48.72%. When the sensitivity was close to 60%, the diagnostic cut-off value of TPO-Ab was 18.6 IU/mL, with a specificity of 97.44%; the diagnostic cut-off value of TG-Ab was 17.36 IU/mL, with a specificity of 94.87%; the diagnostic cut-off value of TT4 was 97.45 nmol/L, with a specificity of 60.26%; and the diagnostic cut-off value of FT4 was 12.71 pmol/L, with a specificity of 58.97%. When >133 IU/mL, TPO-Ab had a sensitivity of 11.34% and a specificity of 98.72% [95% confidence interval (CI): 0.707–0.836]. When >261.1 IU/mL, TG-Ab had a sensitivity of 2.06% and a specificity of 98.72% (95%CI: 0.742–0.864). When >218.25 nmol/L, TT4 had a sensitivity of 1.03% and a specificity of 98.72% (95%CI: 0.567–0.713). When >17.7 pmol/L, FT4 had a sensitivity of 4.12% and a specificity of 98.72% (95%CI: 0.514–0.664) (Tables 3, 4 and Figure 3).

**Table 3:** Analysis of the ROC curves of thyroid function and autoantibodies parameters in the GBS and CIDP groups.

**Variable**	**AUC**	**SE**	**95% CI**	**Youden index**	***P***
TT3	0.600	0.043	0.523–0.673	0.228	0.020
TT4	0.643	0.042	0.567–0.713	0.252	0.000^**^
TSH	0.537	0.044	0.460–0.613	0.135	0.401
FT3	0.528	0.044	0.451–0.603	0.120	0.530
FT4	0.590	0.043	0.514–0.664	0.199	0.038^*^
TPO-Ab	0.776	0.037	0.707–0.836	0.601	0.000^**^
TG-Ab	0.809	0.035	0.742–0.864	0.575	0.000^**^

*The CIDP group was used as the reference group, and the GBS group was used as the measurement group. The differences in TT4, FT4, TPO-Ab, and TG-Ab between the GBS and CIDP groups were statistically significant*,

** *P < 0.01*,

* *P < 0.05*.

**Table 4:** List of cut-off value, sensitivity, and specificity of thyroid function and autoantibodies in GBS and CIDP ROC analysis.

**Variable**	**Criterion**	**Specificity (%)**	**Sensitivity (%)**
TPO-Ab	>13.7 IU/mL^*^	96.15	63.92
	>18.6 IU/mL^#^	97.44	60.82
	>133 IU/mL^Δ^	98.72	11.34
	≤0.01IU/mL^Δ^	97.96	9.09
TG-Ab	>6.6 IU/mL^*^	88.46	69.07
	>17.36 IU/mL^#^	94.87	60.82
	>261.1IU/mL^Δ^	98.72	2.06
	≤0.46 IU/mL^Δ^	98.98	11.69
TT4	>89.15 nmol/L^*^	55.13	70.10
	>97.45 nmol/L^#^	60.26	60.82
	>157.75nmol/L^Δ^	97.44	6.19
	≤69.03nmol/L^Δ^	98.98	6.49
FT4	>12.23 pmol/L^*^	48.72	71.13
	>12.71 pmol/L^#^	58.97	60.82
	>17.7pmol/L^Δ^	98.72	4.12
	≤9.08pmol/L^Δ^	97.96	1.30

* *The diagnostic cut-off value corresponding to the maximum point of the Youden index*.

# *The diagnostic cut-off value corresponds to sensitivity near to 60%*.

Δ *The diagnostic cut-off value corresponds to the maximal specificity*.

**Figure 4:**
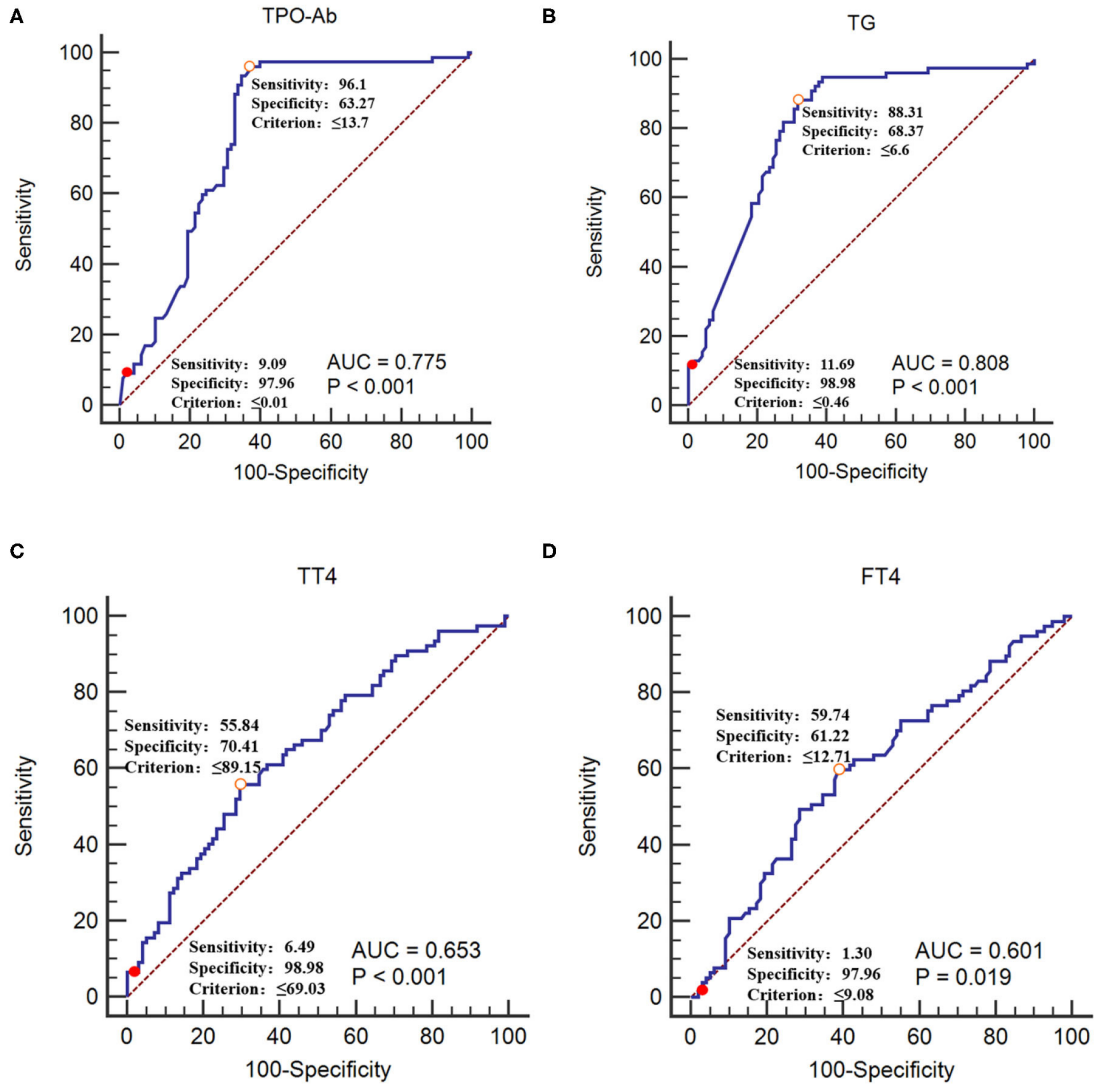
Receiver operating characteristic curves of TPO-Ab, TG-Ab, TT4, and FT4 for CIDP occurrence **(A)** TPO-Ab, **(B)** TG-Ab, **(C)** TT4, and **(D)** FT4. The GBS group was used as the reference group, and the CIDP group was used as the measurement group. 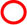 The diagnostic cut-off value corresponds to the maximum point of the Youden index. 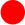 The diagnostic cut-off value corresponds to the maximal specificity.

The GBS group was used as the reference group, and the CIDP group was used as the measurement group. The differences in TT4, FT4, TPO-Ab, and TG-Ab between the GBS and CIDP groups were statistically significant (*P* < 0.05). When lower than 0.01 IU/mL, TPO-Ab had a sensitivity of 9.09% and a specificity of 97.96%. When lower than 0.46 IU/mL, TG-Ab had a sensitivity of 11.69% and a specificity of 98.98%. When lower than 69.03 nmol/L, TT4 had a sensitivity of 6.49% and a specificity of 98.98%. When lower than 9.08 pmol/L, FT4 had a sensitivity of 1.30% and a specificity of 97.96% (Table 4 and Figure 4).

### Logistic Regression Analysis of Risk Factors for GBS Patients With TPO-Ab>133 IU/mL or TG-Ab>261.1 IU/mL

In the GBS and CIDP groups, GBS patients with TPO-Ab>133 IU/mL or TG-Ab>261.1 IU/mL and CIDP patients were used as classification dependent variables, respectively, and general patient data, including sex, age, and levels of TT3, TT4, TSH, FT3, FT4, TPO-Ab, TG-Ab, and HFGS, were used as independent variables for univariate logistic regression analysis. Variables with *P* < 0.05 were included in Multivariate logistic regression analysis. The results of the regression showed that when GBS patients with TPO-Ab>133 IU/mL, the difference in TT3 and TPO-Ab was statistically significant (*P* < 0.05), especially to the TPO-Ab (*P* < 0.01). When GBS patients with TG-Ab>261.1 IU/mL, the difference in TG-Ab was statistically significant (*P* < 0.05). However, the difference in HFGS was not statistically significant (*P* > 0.05, Table 5).

**Table 5:** Logistic regression analysis of risk factors for GBS patients with TPO-Ab>133 IU/mL or TG-Ab>261.1 IU/mL.

**Variable**	**GBS patients with TPO-Ab>133 IU/mL**				**GBS patients with TG-Ab>261.1 IU/mL**			
	**Univariate logistic regression analysis**		**Multivariate logistic regression analysis**		**Univariate logistic regression analysis**		**Multivariate logistic regression analysis**	
	**OR**	***P***	**OR**	***P***	**OR**	***P***	**OR**	***P***
Sex	7.238	0.006**	2.539	0.358	0.000	0.998	–	–
Age	0.977	0.402	–	–	0.925	0.337	–	–
TT3	54.378	0.004**	137.398	0.013^*^	1.316	0.899	–	–
TT4	0.992	0.452	–	–	1.010	0.764	–	–
TSH	0.832	0.316	–	–	0.685	0.108	–	–
FT3	1.402	0.374	–	–	1.329	0.750	–	–
FT4	0.839	0.264	–	–	1.673	0.325	–	–
TPO-Ab	0.986	0.002**	0.989	0.001**	0.994	0.039^*^	0.993	0.054
TG-Ab	0.996	0.113	–	–	0.994	0.014^*^	0.994	0.023^*^
HFGS	1.691	0.400	–	–	0.000	0.999	–	–

GBS patients with TPO-Ab>133 IU/mL or TG-Ab>261.1 IU/mL and CIDP patients were used as classification dependent variables, respectively, and general patient data, including sex, age, and levels of TT3, TT4, TSH, FT3, FT4, TPO-Ab, TG-Ab, and HFGS, were used as independent variables for univariate logistic regression analysis. Variables with P < 0.05 were included in Multivariate logistic regression analysis. The results of the regression showed that when GBS patients with TPO-Ab>133 IU/mL, the difference in TT3 and TPO-Ab was statistically significant (*P < 0.05), especially to the TPO-Ab (**P < 0.01). When GBS patients with TG-Ab>261.1 IU/mL, the difference in TG-Ab was statistically significant,

* *P < 0.05*.

## Discussion

To search for biomarkers between GBS and CIDP, we carried out a data analysis on the disease. Ganglioside antibodies are more closely associated with GBS (10). CIDP is associated with antibodies against the nodes of Ranvier/paranodal NF155 and contactin-1 (CNTN1) antibody (11). In addition to our well-known serum and cerebrospinal fluid immunology-related antibody tests, are there any other serum immune biomarkers that can be easily and quickly tested to identify these diseases? Relevant studies have reported that Fas is a transmembrane receptor involved in the death program of several cell lines, including T lymphocytes (12). Defective Fas function is associated with CIDP, A-CIDP, thyroid autoimmunity (13) development and aggressive evolution, which can be used as a biomarker for the differential diagnosis of GBS and A-CIDP (14). Gautier's research showed that the IL-8 in CSF as a Potential Biomarker in GBS (15). Our study found that increased thyroid function and thyroid autoantibodies are associated with the occurrence of GBS. We envisage the early identification of GBS and CIDP by detecting thyroid autoantibodies because thyroid function evaluation is much easier to obtain, whereas an essay of T cell apoptosis and IL-8 acquisition in CSF are time-consuming and require certain conditions. However, existing Chinese and international publications are mostly case reports, and no clinical studies with large sample sizes have focused on this topic. The pathogenesis of GBS and thyroid autoantibody positivity has not been elucidated and requires further investigation in clinical research.

### The Associations Between Thyroid Autoantibodies (TPO-Ab and TG-Ab) and GBS, CIDP, and Control Groups

The presence of serum thyroid autoantibodies is a major indicator for the diagnosis of HT. TPO-Ab and TG-Ab are very sensitive, and increases in these parameters can confirm HT (16). This study showed that increases in TPO-Ab and TG-Ab were associated with GBS. The TPO-Ab and TG-Ab autoantibody levels of CIDP patients did not differ from those of the control group. These results indicated that the positive rate of thyroid autoantibodies in CIDP patients was similar to that of the control population, although we did not consider the possibility of elevated thyroid autoantibody levels in the general population. There was no significant difference in HFGS score (representing disease severity) between the GBS and CIDP groups. Therefore, we concluded that there was no significant correlation between the increase of thyroid functions and autoantibody levels and disease severity. In logistic regression analysis, TT3 and TPO-Ab was a risk factor for GBS patients with TPO-Ab was higher than 133 IU/mL, especially to the TPO-Ab.TG-Ab was a risk factor for GBS patients with TG-Ab higher than 261.1 IU/mL. However, HFGS is not a risk factor. This study showed that the differences in thyroid autoantibody are not driven by severity. Our ROC curve analysis showed that TPO-Ab higher than 133IU/mL or lower than 0.01 IU/mL and TG-Ab higher than 261.1 IU/mL or lower than 0.46 IU/mL had high specificity for differentiation between GBS and CIDP and could be used as an auxiliary reference indictor for differentiation between the two diseases. HT with comorbid GBS may be caused by dysregulation of the immune system, which may be different from the pathophysiology underlying HT with comorbid CIDP; the possible mechanisms are analyzed below.

The possible mechanism underlying the increase in thyroid autoantibodies in GBS patients may be a consequence of an immunological interaction between autoantibodies and neuronal cells (6). Indeed, ganglioside antibodies play an important role in the possible mechanism. Patients with positive thyroid autoantibodies had significantly increased responses to ganglioside antibodies (17). Ganglioside is an important link between pathogens and nerve tissue components in the pathogenesis of GBS. The production of ganglioside antibodies interferes with myelination and damages the integrity of myelin sheaths to beget GBS, while CIDP lacks pathogenic infection and other relevant links. Whether it is precursor infection that causes the thyroid autoantibodies titres in GBS to significantly increase needs further discussion. In addition to the effect of ganglioside antibodies on the integrity of myelin sheaths, cytokines [such as interferon-γ, tumor necrosis factor α (TNF-α), and interleukin 17 (IL-17)] (18, 19) and oxidative stress [such as the production of large amounts of reactive oxygen species (ROS) in cells] (20, 21) were also directly involved in the pathological process of demyelination in patients with GBS and CIDP, which are also associated with the occurrence of autoimmune thyroid disease and elevated TPO-Ab antibody levels (22, 23). Moreover, a specific genetic background may cause shared susceptibility to these autoimmune diseases (24). Although GBS and CIDP both belong to immune-mediated peripheral nerve-demyelinating diseases, they have long been regarded differently in the disease spectrum. As mentioned above, defective Fas function has resulted in the simultaneous occurrence of CIDP and autoimmune thyroid diseases (13). Thus, the possibility of common susceptibility between CIDP and HT is taken into account. However, are GBS and HT more susceptible? To date, no relevant reports have been issued. Further study is necessary to explore whether the possible mechanism that promotes GBS and the increase in thyroid autoantibodies differ from those that act in CIDP.

### The Associations Between Thyroid Function (TT3, TT4, FT3, FT4, and TSH) and GBS, CIDP, and Control Groups

Most T4 binds with its transport proteins (thyroxine-binding globulin (TBG), prealbumin, and albumin). Changes in the TBG-binding ability and the concentration of T4 under physiological and pathological conditions influence TT4. This study showed that the TT4 and FT4 levels in GBS patients were higher than those in CIDP and Control group patients, especially the TT4 level. The existence of a certain step in the disease course of GBS that affects the binding ability of TBG and leads to an increase in TT4 cannot be ruled out, which still requires further clinical studies for confirmation.

### The Associations Between Sex Differences and GBS and CIDP

Autoimmune thyroid diseases are more common in women, but the incidence and prevalence of GBS among men are both increasing, and genetic and environmental factors likely play a role in this trend (25). The GBS and CIDP groups both included more men than women in this study, which is consistent with the increasing trend of GBS among men mentioned above, but no reports indicate that CIDP is more common in men.

A limitation of this study is its retrospective design, and we could not ensure that each patient received complete examinations, such as thyroid color Doppler ultrasound and anti-ganglioside antibody detection. Furthermore, the Medical Research Council (MRC) sum score was not assessed for each enrolled patient; therefore, the correlation between the MRC sum score and increased thyroid autoantibodies levels could not be evaluated. In the future, prospective studies should be performed with complete examinations to improve the accuracy of the results.

In summary, our study indicated that an elevation of thyroid autoantibodies was associated with GBS. Among the GBS patients, TPO-Ab had higher auxiliary diagnostic value for male GBS patients. The use of thresholds of higher than 13.7 IU/mL for TPO-Ab or higher than 6.6 IU/mL for TG-Ab yielded the best sensitivity and specificity for differentiating GBS from CIDP. When TPO-Ab was higher than 133 IU/mL and TG-Ab was higher than 261.1 IU/mL, a diagnosis of GBS was almost certain. However, when TPO-Ab was lower than 0.01 IU/mL and TG-Ab was lower than 0.46 IU/mL, a diagnosis of CIDP was almost certain. TPO-Ab and TG-Ab may be used as biomarkers for the differential diagnosis of GBS and CIDP. Early detection of thyroid autoantibodies abnormalities in patients has important clinical value for predicting GBS.

## Data Availability Statement

All datasets presented in this study are included in the article/Supplementary Material.

## Ethics Statement

The studies involving human participants were reviewed and approved by Ethics committee of Beijing tiantan hospital affiliated to capital medical university. Written informed consent from the participants' legal guardian/next of kin was not required to participate in this study in accordance with the national legislation and the institutional requirements.

## Author Contributions

YT managed and collected data and wrote the manuscript. XG researched data and wrote the manuscript. XY conceived and designed the study and contributed to the manuscript. GZ, WZ, and ZL researched the data, contributed to the discussion, and reviewed and edited the manuscript. All authors contributed to the article and approved the submitted version.

## Conflict of Interest

The authors declare that the research was conducted in the absence of any commercial or financial relationships that could be construed as a potential conflict of interest.
